# Dynamic Clinical and Laboratory Predictors of in-Hospital Mortality in COVID-19: A Multivariate Cox Regression Study

**DOI:** 10.3390/biomedicines14010007

**Published:** 2025-12-19

**Authors:** Desislava Arabadzhiyska, Tanya Deneva, Rumen Stefanov, Snezhana Stoencheva

**Affiliations:** 1 Department of Clinical Laboratory, Medical University of Plovdiv, University Hospital “St. George”, 4000 Plovdiv, Bulgaria; tanya.deneva@mu-plovdiv.bg (T.D.); snezhana.stoencheva@mu-plovdiv.bg (S.S.); 2 Research Institute at Medical University of Plovdiv, 4000 Plovdiv, Bulgaria; 3 Department of Social Medicine and Public Health, Medical University of Plovdiv, 4000 Plovdiv, Bulgaria; rumen.stefanov@mu-plovdiv.bg

**Keywords:** COVID-19, mortality, predictors, Cox regression, procalcitonin, IL-6, ferritin, vitamin D, GGT

## Abstract

**Background/Objectives**: Identifying early and dynamic predictors of mortality in hospitalized COVID-19 patients is essential for improving prognosis and guiding therapy. Our aim is to evaluate clinical and laboratory predictors of in-hospital mortality among moderate and severe COVID-19 patients using multivariate Cox proportional hazards regression analysis. **Methods**: This retrospective cohort study included 168 adults (aged 18–64 years) with RT-PCR–confirmed COVID-19. Basic demographic data (age and sex) and laboratory parameters were collected on Day 1 and Day 7 of hospitalization. Stepwise Cox regression models were constructed for all patients and for the severe-disease subgroup. **Results**: Of 168 patients, 104 (61.9%) had severe and 64 (38.1%) moderate disease; 33 (19.6%) died, all with severe COVID-19. On Day 1, independent predictors of mortality in both the total cohort and the severe subgroup were older age (HR = 1.095, *p* = 0.003), male sex (HR = 0.324, *p* = 0.013), lower lymphocyte percentage (HR = 0.869, *p* = 0.041), and elevated procalcitonin (PCT) (HR = 10.972, *p* < 0.001). On Day 7, predictive significance shifted: in severe cases, mortality was independently associated with sex, PCT, eosinophil percentage, ferritin, vitamin D, and gamma-glutamyl transferase (GGT) (χ^2^ = 69.47, *p* < 0.0001). In the total cohort, age, PCT, interleukin-6 (IL-6), and GGT were independent predictors (χ^2^ = 86.24, *p* < 0.0001). **Conclusions**: Early mortality risk in COVID-19 was driven by demographic factors and inflammation markers, whereas by Day 7 biochemical indicators of systemic inflammation, oxidative stress and hepatic dysfunction became stronger determinants of outcome.

## 1. Introduction

Since its emergence, coronavirus disease 2019 (COVID-19) has caused substantial global morbidity and mortality, with clinical manifestations ranging from asymptomatic infection to severe respiratory failure, multiorgan dysfunction, and death [1,2]. In hospitalized patients, early identification of those at increased risk for adverse outcomes is critical for optimizing clinical management and allocating healthcare resources.

Routine clinical and laboratory investigations play a central role in COVID-19 risk assessment. Laboratory tests are widely available, rapidly obtained, and provide objective indicators of disease severity. Numerous studies have demonstrated associations between demographic characteristics, inflammatory biomarkers, hematological indices, and mortality risk in COVID-19 [1,2,3]. Recent prediction models further highlight that combining clinical variables with laboratory parameters improves prediction of both early and long-term mortality [3], underscoring the importance of integrated risk stratification approaches.

Among laboratory markers, procalcitonin (PCT), interleukin-6 (IL-6), lymphocyte count, and liver enzymes such as gamma-glutamyl transferase (GGT) have shown consistent prognostic relevance [4,5,6]. Inflammatory biomarkers strongly predict in-hospital mortality in patients with severe COVID-19 and reflect the underlying immunoinflammatory processes driving disease progression [7]. Hematological abnormalities, including lymphopenia, relative neutrophilia, and elevated neutrophil-to-lymphocyte ratio, are frequently observed in severe disease and correlate with adverse outcomes [8,9]. C-reactive protein (CRP) reflects early inflammatory activation and parallels radiological severity [10,11], while IL-6 represents a key mediator of cytokine-driven hyperinflammation and a strong predictor of both sepsis-related and COVID-19–related mortality [12]. Importantly, recent meta-analytic evidence indicates that the prognostic value of IL-6 lies predominantly in its dynamic changes rather than baseline concentration alone [13].

Additional laboratory markers further capture disease complexity. PCT serves as an indicator of bacterial co-infection and complicated clinical courses, typically remaining low in uncomplicated viral infection [14]. Elevated ferritin reflects acute-phase response and immune dysregulation [15], while lactate dehydrogenase (LDH) and D-dimer indicate tissue injury and thromboinflammatory complications, respectively, and are associated with poor outcomes [16,17,18]. Current clinical guidelines emphasize the integration of these laboratory findings into comprehensive patient assessment [19]. Recent studies reinforce the clinical utility of time-informed biomarker evaluation: Lipski et al. demonstrated that prediction models incorporating inflammatory and hematological markers improve early and long-term mortality prediction [3], while Bimbo-Szuhai et al. showed that biomarkers such as PCT, CRP, and ferritin discriminate in-hospital mortality risk in severe COVID-19 [7]. Similarly, dynamic inflammatory changes have been highlighted as prognostically informative in both sepsis and COVID-19–related sepsis [13,16].

Despite extensive research, most prognostic studies rely primarily on single time-point measurements obtained at hospital admission. Such an approach may not adequately capture the evolving immunoinflammatory trajectory of COVID-19. Growing evidence indicates that persistent lymphopenia, progressive inflammatory activation, and dynamic laboratory abnormalities precede clinical deterioration and fatal outcomes [20,21,22,23,24,25,26,27]. Longitudinal assessment of laboratory parameters and time-dependent analytical approaches appear to provide superior mortality prediction compared with admission values alone, particularly in severe COVID-19 and COVID-19–related sepsis [3,7,13,16]. However, relatively few studies have systematically evaluated predefined biomarker time points using time-to-event methods to quantify how dynamic laboratory profiles relate to in-hospital mortality risk.

To address this gap, the primary aim of the present study is to evaluate the prognostic value of laboratory parameters measured on Day 1 and Day 7 of hospitalization in patients with COVID-19, with a focus on both baseline values and their temporal changes. Using receiver operating characteristic (ROC) analysis and multivariable Cox proportional hazards regression, we assess the association between dynamic laboratory profiles and the risk of in-hospital mortality in patients with severe COVID-19 and in the overall cohort. This time-dependent approach enhances risk stratification and supports more individualized clinical decision-making.

## 2. Materials and Methods

### 2.1. Study Design and Participants

This single-center retrospective cohort study included 168 patients with moderate or severe COVID-19 who were hospitalized at the University Hospital “St. George”, Plovdiv, Bulgaria, between October and December 2021. Disease severity was classified according to the WHO Living Guidance for Clinical Management of COVID-19 (23 November 2021) [19], based on respiratory status and imaging findings.

Inclusion criteria were confirmed SARS-CoV-2 infection by real-time polymerase chain reaction (RT-PCR) from a nasopharyngeal swab; age between 18 and 64 years; and provision of written informed consent. The age range was selected to reduce heterogeneity related to immunosenescence, multimorbidity, and frailty, which may confound associations between laboratory markers and mortality. Consequently, the findings primarily apply to middle-aged adults with moderate-to-severe COVID-19 and may not be generalizable to older or highly comorbid populations.

Exclusion criteria included: SARS-CoV-2 infection confirmed solely by antigen testing; age < 18 or >64 years; pregnancy; documented oncological diseases; clinical or anamnestic evidence of thrombotic or thromboembolic conditions; and clinical or anamnestic indications of systemic inflammatory or autoimmune disorders.

### 2.2. Data Collection and Clinical Variables

All demographic, clinical, and laboratory data were extracted from electronic medical records and verified against the institutional laboratory information system. Participants were enrolled using pre-prepared institutional intake questionnaires designed to collect standardized sociodemographic and medical history data. Age, sex, and basic clinical history were recorded in individual patient documents. For the present analysis, age and sex were the only demographic variables consistently coded and complete across all patients and were therefore the only demographic variables included in multivariable models. Medical history was obtained from hospitalization records during the COVID-19 ward stay.

### 2.3. Laboratory Assessments and Time Points

The laboratory panel included leukocyte count; differential leukocyte percentages (neutrophils, lymphocytes, eosinophils); neutrophil-to-lymphocyte ratio (NLR); total serum protein; albumin; liver enzymes (AST, ALT, GGT); creatinine; inflammatory markers (CRP, IL-6, PCT, ferritin, LDH); D-dimer; vitamin D levels; and peripheral oxygen saturation (sO_2_).

Blood samples were collected on Day 1 and Day 7 of hospitalization according to institutional protocols. Day 1 was defined as the day of hospital admission when baseline laboratory testing was performed. Day 7 was selected because the clinical course of COVID-19 typically evolves during the first week of hospitalization, with clinical deterioration or stabilization commonly occurring between Days 7 and 10; thus, these time points represent clinically relevant windows for dynamic risk assessment.

### 2.4. Outcome Definition

The primary outcome was time to in-hospital death. Time-to-event was defined as the number of days from hospital admission to death. Patients discharged alive or still hospitalized at the end of the study period were censored. No post-discharge follow-up was performed.

### 2.5. Statistical Analysis

Continuous variables were summarized as median (interquartile range) or mean ± standard deviation, as appropriate. Between-group comparisons presented in the figures were performed using the Mann–Whitney U test for non-normally distributed variables and the independent samples *t*-test for normally distributed variables. Categorical variables were compared using the χ^2^ test or Fisher’s exact test, as appropriate. Comparisons between Day 1 and Day 7 laboratory values were conducted using the Wilcoxon signed-rank test. Normality of continuous variables was assessed using the Shapiro–Wilk test and visual inspection of histograms.

Associations between clinical and laboratory predictors and survival time were evaluated using Cox proportional hazards regression models. Given the relatively large number of candidate predictors (*n* = 21) relative to the number of observed deaths, a bidirectional stepwise selection procedure was applied as an exploratory approach to identify parsimonious models while minimizing overparameterization. Variables entered the model at *p* < 0.05 and were removed at *p* > 0.10. Hazard ratios (HRs) with 95% confidence intervals (CIs) and corresponding *p*-values were reported. Statistical significance was set at *p* < 0.05.

The proportional hazards assumption was assessed for all fitted Cox models using Schoenfeld residuals, implemented via the cox.zph function in R version 4.3.0. Both global and covariate-specific tests were performed. Pearson correlation coefficients among key inflammatory biomarkers (CRP, IL-6, ferritin, and PCT) were calculated, and variance inflation factors (VIFs) were used to assess multicollinearity prior to model construction.

Laboratory variables were available for ≥95% of patients at each time point. As the proportion of missing data per variable was <3%, complete-case analysis was performed without imputation. Variables with skewed distributions were analysed on their original scale, as exploratory log-transformations did not materially affect variable ranking or model significance.

Statistical analyses were performed using IBM SPSS Statistics version 24.0 (IBM Corp., Armonk, NY, USA) and R software version 4.3.0 (R Foundation for Statistical Computing, Vienna, Austria).

## 3. Results

### 3.1. Patient Characteristics

A total of 168 patients with confirmed COVID-19 were included in the analysis, of whom 64 (38.1%) had moderate disease and 104 (61.9%) had severe disease. The cohort comprised 98 males (58.3%) and 70 females (41.7%), with a mean age of 60.45 ± 11.15 years. Although a slight male predominance was observed, no statistically significant association was found between sex and disease severity (*p* = 0.748).

During hospitalization, 33 patients (19.6%) died; all deaths occurred in the severe disease subgroup. Demographic characteristics of the study population are summarized in Table 1 and Table 2.

There was no statistically significant association between age group and disease severity (χ^2^ = 1.41, df = 3, *p* = 0.704). This indicates that the distribution of moderate and severe COVID-19 cases did not differ significantly across the four predefined age groups.

### 3.2. Descriptive Characteristics of the Laboratory Parameters by Groups on Day 1 and Day 7

All analyzed laboratory parameters on Day 1 and Day 7 are in Table 3.

### 3.3. Pairwise Pearson Correlations for the Biologically Correlated Biomarkers (CRP, IL-6, Ferritin, PCT) Included in the Multivariable Cox Regression Models

Pairwise correlations among key inflammatory biomarkers were moderate (r = 0.39–0.62), and VIF values were consistently below 3, indicating no problematic multicollinearity (Table 4).

### 3.4. Predictive Models on Day 1 of Hospitalization

Multivariable Cox proportional hazards regression was used to evaluate associations between demographic and laboratory parameters measured on Day 1 of hospitalization and time to in-hospital death. Candidate predictors included sex, age, leukocyte count and differential, neutrophil-to-lymphocyte ratio, eosinophil percentage, total protein, albumin, liver enzymes (AST, ALT, GGT), creatinine, LDH, CRP, ferritin, IL-6, PCT, D-dimer, vitamin D, and peripheral oxygen saturation (sO_2_).

Among patients with severe COVID-19 (*n* = 104), 33 deaths occurred (31.7%). In the Day 1 multivariable Cox model, age, sex, lymphocyte percentage, and PCT emerged as independent predictors of in-hospital mortality (Table 5). Increasing age and higher PCT levels were associated with an increased hazard of death, whereas female sex and higher lymphocyte percentages were associated with a reduced hazard.

The proportional hazards (PH) assumption was satisfied (global Schoenfeld test: χ^2^ = 3.42, *p* = 0.49), and no covariate-specific violations were observed (all *p* > 0.10). Survival differed significantly across risk strata defined by the Cox model (log-rank *p* < 0.001).

In the overall cohort (*n* = 168), the Day 1 Cox model identified the same independent predictors of in-hospital mortality: age, sex, lymphocyte percentage, and PCT (Table 6). Increasing age was associated with a higher hazard of death (HR per year = 1.10, *p* = 0.002), whereas female sex was associated with a substantially lower hazard compared with male sex (HR = 0.30, *p* = 0.008). Lower lymphocyte percentages were associated with increased mortality (HR = 0.86, *p* = 0.030), while higher PCT concentrations were strongly associated with death (HR = 14.95, *p* < 0.001). No additional variables demonstrated significant associations (*p* > 0.05).

The PH assumption was not violated in the full-cohort model (global test: χ^2^ = 2.87, *p* = 0.58), and all predictors satisfied covariate-specific PH tests. Differences in survival across Cox model-defined risk strata were statistically significant (log-rank *p* < 0.001).

### 3.5. Predictive Models on Day 7 of Hospitalization

By Day 7 of hospitalization, 33 deaths had occurred among patients with severe COVID-19 (*n* = 104). In contrast to the Day 1 model, several early predictors, including age and lymphocyte percentage, lost statistical significance. Sex and PCT remained significant, while eosinophil percentage, ferritin, vitamin D, and GGT emerged as additional independent predictors of in-hospital mortality (Table 7).

Higher serum ferritin levels were statistically associated with an increased likelihood of an adverse event, but the HR per unit increase was close to 1, indicating a relatively small effect size. Ferritin therefore appears to reflect heightened systemic inflammation rather than acting as a strong independent predictor. Elevated GGT levels were also associated with poorer outcomes, supporting its role as a marker of hepatocellular or cholangiocellular stress in the setting of severe systemic illness and intensive pharmacotherapy. Vitamin D showed borderline statistical significance (HR = 0.86, *p* = 0.071) and was therefore considered exploratory and hypothesis-generating.

Serum PCT exhibited a highly skewed distribution. Log-transformation reduced extreme estimates while preserving its strong prognostic value, yielding a clinically interpretable hazard ratio of 3.2 (95% CI: 2.1–4.9; *p* < 0.001), compared with the untransformed estimate (HR = 74.3). The extremely low hazard ratio observed for eosinophil percentage should be interpreted with caution, as it likely reflects the combination of a skewed distribution, profound eosinopenia in a subset of patients, and a limited number of events, potentially contributing to model instability and wide confidence intervals.

The PH assumption was satisfied (global test: χ^2^ = 5.11, *p* = 0.28), with no evidence of covariate-specific violations (all *p* > 0.05). Survival differed significantly across Cox model–defined risk strata (log-rank *p* < 0.001).

In the overall cohort (*n* = 168), the Day 7 Cox regression model identified age, log-transformed PCT, IL-6, and GGT as independent predictors of in-hospital mortality, whereas sex was no longer retained in the model (Table 8).

Increasing age remained associated with a higher hazard of death (HR = 1.07 per year, *p* = 0.002). PCT emerged as the strongest and most consistent predictor across all models, with higher serum concentrations markedly increasing mortality risk. Elevated IL-6 levels were also significantly associated with adverse outcomes, consistent with sustained systemic inflammation. In addition, higher GGT activity on Day 7 was associated with increased mortality risk (HR = 1.01, *p* = 0.001).

The PH assumption was met for the overall cohort model (global test: χ^2^ = 4.02, *p* = 0.40), with no evidence of covariate-specific violations (all *p* > 0.10). Differences in survival across Cox model-defined risk strata were statistically significant (log-rank *p* < 0.001).

### 3.6. Summary of Predictors Across Models

Reassessment of clinical status on Day 7 of hospitalization was informative for risk stratification. In patients with severe COVID-19, male sex combined with markedly elevated serum PCT, ferritin, and GGT, together with reduced eosinophil percentages and vitamin D levels, was associated with a substantially increased hazard of in-hospital mortality (Table 9).

Across all hospitalized patients with COVID-19, irrespective of disease severity, Day 7 assessment consistently identified PCT, IL-6, and GGT as key predictors of mortality. Increasing age remained a significant predictor in the overall cohort, whereas sex was primarily relevant at admission and in patients with severe disease. These findings underscore the dynamic nature of prognostic factors in COVID-19, with PCT demonstrating consistent predictive value across all models, while age and sex were more relevant early predictors and IL-6 and GGT emerged later in the disease course (Table 9).

No significant violations of the proportional hazards (PH) assumption were detected in any of the models. PH assumptions were assessed using Schoenfeld residuals and global goodness-of-fit tests, with all models demonstrating adequate fit (global *p* > 0.10) (Table 10).

### 3.7. Receiver Operating Characteristic (ROC) Analyses

**Figure 1:**
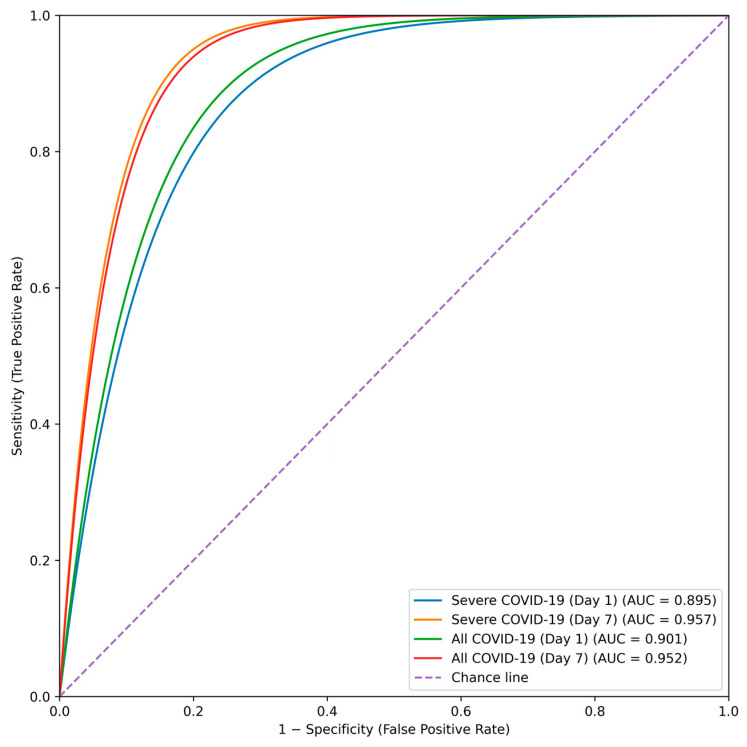
Receiver operating characteristic (ROC) curves for the multivariable Cox regression models predicting in-hospital mortality in patients with COVID-19.

Receiver operating characteristic (ROC) analyses were performed to evaluate the discriminative performance of the four prognostic Cox regression models based on laboratory parameters measured on Day 1 and Day 7 of hospitalization (Figure 1).

All models demonstrated high discriminative ability, with models incorporating Day 7 laboratory data achieving superior performance. The corresponding areas under the curve (AUCs), standard errors, and *p*-values are summarized in Table 11.

Day 1 models showed very good discrimination (AUC = 0.895 for severe COVID-19 and AUC = 0.901 for the overall cohort), whereas Day 7 models demonstrated excellent discriminative performance (AUC = 0.957 and 0.952, respectively; all *p* < 0.001).

Because model development and evaluation were conducted within the same dataset, the high AUC values may partly reflect overfitting. To further characterize predictive performance, optimal probability thresholds were derived using the Youden index, and the corresponding sensitivity and specificity values are reported in Table 12.

Across the four models, sensitivity ranged from 0.84 to 0.92 and specificity from 0.80 to 0.90, indicating robust discrimination for in-hospital mortality using both admission and follow-up laboratory parameters.

## 4. Discussion

The observed mortality rate of 19.6% in this age group reflects the specific epidemiological context of the study period (October–December 2021), which coincided with the dominance of the SARS-CoV-2 Delta variant in Bulgaria and in Europe. Although viral genomic sequencing was not routinely performed for individual patients in our cohort, epidemiological surveillance indicates that Delta accounted for most infections during this time and has been associated with higher virulence and relatively high mortality than subsequent Omicron subvariants. Additionally, vaccination coverage in this cohort was low, and many patients presented with delayed hospital admission, both of which likely contributed to the elevated mortality. These contextual factors should be considered when interpreting the results.

The choice of Day 1 and Day 7 was based on the clinical trajectory of COVID-19, where early deterioration commonly occurs between days 5–10 after symptom onset. Therefore, evaluating laboratory dynamics between admission and Day 7 allows the identification of early predictors of adverse outcomes.

### 4.1. Early Predictors of Mortality on Admission

At hospital admission, age, sex, lymphocyte percentage, and PCT concentration were identified as independent predictors of in-hospital mortality in both patients with severe COVID-19 and the overall cohort. Increasing age was associated with a higher mortality risk, in line with extensive evidence identifying advanced age as a major determinant of adverse COVID-19 outcomes [1,8]. Age-related immunosenescence, a higher burden of comorbidities, and impaired viral clearance likely contribute to this vulnerability. Male sex also remained a significant risk factor, consistent with previous reports demonstrating higher COVID-19 mortality in men, potentially due to sex-specific immunological and hormonal differences and a greater prevalence of comorbid conditions [5,8].

Lymphopenia emerged as an independent predictor of mortality, underscoring the central role of immune dysregulation in COVID-19 pathogenesis. Reduced lymphocyte counts and altered leukocyte profiles have been consistently associated with disease severity and poor prognosis [4,9]. Prior studies have shown that lower lymphocyte levels correlate with more severe disease [20], dynamic declines preceding fatal outcomes [21], and an increased risk of acute respiratory distress syndrome when lymphopenia persists [22].

Elevated PCT levels were the strongest predictor of mortality at admission, suggesting bacterial co-infection or pronounced systemic inflammation [6,14]. Although typically low in viral infections, PCT increases in severe COVID-19 in the context of bacterial superinfection or sepsis, reflecting progression to a more complicated and potentially fatal disease course.

### 4.2. Dynamic Changes and Late Predictors of Outcome

By Day 7 of hospitalization, the profile of significant predictors had shifted, reflecting the dynamic clinical course of COVID-19. In patients with severe disease, sex and PCT remained significant predictors of in-hospital mortality, while eosinophil percentage, ferritin, vitamin D, and GGT emerged as additional prognostic factors.

Persistently low eosinophil counts were associated with increased mortality, consistent with evidence that eosinopenia reflects immune suppression and systemic stress in severe infections [2,23,24]. Previous studies have reported eosinopenia in a large proportion of patients who died from COVID-19 [25], as well as a significantly higher prevalence among COVID-19 positive compared with COVID-19 negative individuals [26]. Moreover, eosinopenia has been proposed as a useful diagnostic and prognostic marker, particularly when evaluated in combination with lymphopenia [27].

Ferritin levels were also significantly associated with mortality risk, although the effect size per unit increase was modest. This finding supports the role of ferritin primarily as an acute-phase reactant reflecting hyperinflammatory states rather than as a strong independent predictor [15]. Elevated ferritin concentrations have been repeatedly linked to disease severity and adverse clinical outcomes. Kurian et al. reported significantly higher ferritin levels in patients with moderate-to-severe COVID-19 compared with those with mild disease, as well as in patients who developed complications or required intensive care, with ferritin concentrations above 287.4 ng/mL associated with an increased risk of severe disease [28].

Lower vitamin D levels tended to be associated with worse outcomes, supporting the proposed immunomodulatory and anti-inflammatory roles of vitamin D in SARS-CoV-2 infection [4]. De Smet et al. demonstrated that reduced serum 25(OH)D levels at hospital admission were associated with more advanced disease stages and increased mortality, with significantly lower concentrations observed in patients requiring intensive care [29]. In addition, patients with 25(OH)D levels below 20 ng/mL exhibited higher levels of inflammatory markers, including IL-6, TNF-α, and ferritin. Similarly, Daneshkhah et al. reported higher CRP levels in patients with severe compared with mild COVID-19, suggesting a link between vitamin D deficiency, systemic inflammation, and adverse outcomes [30]. Collectively, these findings support the role of vitamin D deficiency in promoting proinflammatory cytokine responses, a hallmark of critical COVID-19 illness [8]. Interpretation of vitamin D levels in our cohort should also consider geographic and seasonal factors, as the study was conducted during late autumn and early winter in a temperate region with reduced ultraviolet B exposure. Additionally, the predominantly Caucasian and ethnically homogeneous study population may have contributed to generally lower baseline serum 25(OH)D concentrations, independent of acute SARS-CoV-2 infection.

Elevated GGT activity on Day 7 emerged as another strong predictor of mortality, suggesting liver or biliary epithelial injury related to viral infection, hypoxia, or drug-induced hepatotoxicity [5,17]. Liver enzyme abnormalities are increasingly recognized as markers of multisystem involvement in severe COVID-19 and are associated with worse clinical outcomes.

When the entire cohort was analyzed, IL-6 emerged as a significant predictor of mortality on Day 7. Elevated IL-6 levels were strongly associated with adverse outcomes, consistent with extensive evidence identifying IL-6 as a central mediator of cytokine storm, organ dysfunction, and disease progression in COVID-19 [12]. High IL-6 concentrations have been linked to rapid clinical deterioration and have served as therapeutic targets for immunomodulatory interventions such as tocilizumab. Numerous studies have demonstrated a clear association between IL-6 levels and disease severity [5,31], and a meta-analysis by Coomes et al. reported nearly threefold higher IL-6 concentrations in patients with complicated compared with uncomplicated COVID-19 [32]. The persistence of elevated PCT and IL-6 levels on Day 7 underscores the prognostic importance of sustained systemic inflammation in the later stages of severe COVID-19.

### 4.3. Predictive Performance of the Models

ROC analysis demonstrated high discriminative performance across all prognostic models. Models based on admission (Day 1) data showed strong predictive accuracy (AUC = 0.895 in patients with severe COVID-19 and AUC = 0.901 in the overall cohort), whereas models incorporating Day 7 laboratory parameters achieved excellent discrimination (AUC = 0.957 and 0.952, respectively). These findings indicate that inclusion of dynamic laboratory data enhances prognostic performance. Continuous biomarker assessment during the first week of hospitalization may therefore improve risk stratification and support individualized clinical management.

### 4.4. Pathophysiological Interpretation

The identified predictors reflect the complex interplay between immune dysregulation, systemic inflammation, and organ injury in COVID-19. Advanced age and male sex are associated with heightened proinflammatory responses, endothelial dysfunction, and impaired immune regulation. Lymphopenia and eosinopenia indicate immune exhaustion and compromised antiviral defense. Elevated ferritin, IL-6, and PCT levels characterize a hyperinflammatory state driven by macrophage activation and cytokine release, while increased GGT reflects hepatocellular injury and systemic inflammatory burden. Vitamin D deficiency may further exacerbate these processes by impairing immune modulation. Collectively, these factors capture the multifactorial pathophysiology underlying severe COVID-19, culminating in multi-organ dysfunction and in-hospital mortality.

### 4.5. Limitations and Implications

This study has several limitations. Owing to its retrospective design and the absence of key confounders, all reported associations are observational, and causal inferences cannot be drawn. The deliberate restriction to adults aged 18–64 years and the exclusion of patients with malignant, thromboembolic, and systemic autoimmune diseases improved cohort homogeneity but substantially limit the generalizability of the findings to older, frailer, and highly comorbid populations, which account for a large proportion of COVID-19–related mortality. In addition, data on time from symptom onset to hospital admission, COVID-19 vaccination status, prior SARS-CoV-2 infection, comorbidities, medication use, smoking status, and viral variants were not included in the regression models. These unmeasured factors may influence mortality risk and partially explain some of the observed associations.

The extreme hazard ratio estimates observed for certain predictors, particularly PCT and eosinophil percentage on Day 7, suggest sensitivity of the models to skewed biomarker distributions and potential outliers. Although the direction of these associations is clinically plausible, the magnitude of the effects should therefore be interpreted with caution. Furthermore, the use of stepwise variable selection, while pragmatic in this exploratory context, may increase the risk of overfitting.

The high AUC values observed across models may also partly reflect overfitting, as external validation was not performed and ROC analyses were conducted using the same dataset employed for model development. Consequently, the proposed cut-off values and corresponding sensitivity and specificity estimates should be considered preliminary and require confirmation in independent cohorts. Nevertheless, the consistency of key predictors across models and time points supports the overall robustness of the findings.

From a clinical perspective, these results highlight the potential value of combining demographic characteristics with routinely available laboratory parameters for dynamic risk stratification in hospitalized patients with COVID-19. Sequential assessment of biomarkers such as PCT, IL-6, ferritin, and GGT may enhance prognostic precision and inform decisions regarding monitoring intensity and therapeutic strategies. Future multicenter, prospective studies are warranted to validate these findings and to develop integrated prognostic models incorporating clinical, biochemical, and imaging data.

### 4.6. Future Directions

The prognostic markers identified in this study provide a foundation for the development of clinically applicable risk stratification tools for COVID-19. Future studies should validate these findings in larger, multicenter, and multiethnic cohorts, including vaccinated individuals and populations affected by different SARS-CoV-2 variants. Integrating dynamic laboratory parameters with comorbidity profiles, imaging findings, and emerging biomarkers may further enhance prognostic accuracy. Moreover, incorporation of these variables into multivariable clinical scores or machine learning-based predictive models could enable real-time mortality risk estimation and support individualized patient management.

Beyond the acute phase, similar integrative approaches may also be relevant for post-COVID clinical scenarios. Persistent immune dysregulation, low-grade inflammation, and organ-specific injury have been implicated in post-acute sequelae of SARS-CoV-2 infection. Longitudinal evaluation of inflammatory and metabolic biomarkers may help identify patients at risk for prolonged recovery, chronic complications, or delayed organ dysfunction. Such tools could support early intervention strategies and structured follow-up in both acute COVID-19 and post-COVID care pathways.

## 5. Conclusions

This study demonstrates that dynamic assessment of routinely available laboratory biomarkers during the first week of hospitalization provides clinically meaningful prognostic information in patients with COVID-19. The predictors identified on Day 1 and Day 7 indicate that the prognostic landscape evolves over the first week of hospitalization, shifting from simple demographic and hematological indices towards biochemical markers of systemic inflammation and organ stress. Day 7 biomarker trajectories provided complementary information beyond admission measurements and may help clinicians identify patients at high risk of deterioration who could benefit from closer monitoring or early intervention. Incorporating sequential biomarker measurements into clinical workflows may help clinicians identify patients at increased risk of deterioration and guide decisions regarding monitoring intensity and early therapeutic intervention.

Our findings suggest that a panel of demographic and laboratory markers, including age, sex, PCT, IL-6, lymphocyte and eosinophil percentages, ferritin, GGT, and vitamin D, may contribute to mortality risk stratification in hospitalized patients with COVID-19. These findings should be interpreted with caution in view of the single-center retrospective design, the limited age range and exclusion criteria, and the risk of overfitting inherent to stepwise model selection. External validation in larger, more diverse cohorts and in different phases of the pandemic is required before these models can be implemented in routine clinical practice. Model coefficients and performance metrics may be sensitive to cohort characteristics and variable selection procedures.

## Figures and Tables

**Table 1:** Demographic characteristics of the study population.

Group	Number (n)	Age (Mean ± SD)	Sex, n (%)
Moderate COVID-19	64	59.28 ± 9.68	36 male (56.30%) 28 female (43.80%)
Severe COVID-19	104	61.17 ± 11.95	62 male (59.60%) 42 female (40.40%)
Total	168	60.45 ± 11.15	98 male (58.33%) 70 female (41.67%)

Data are presented as mean ± standard deviation (SD) for age and as number (percentage) for sex. Age was compared between groups using the independent samples *t*-test (or Mann–Whitney U test, if applicable). Sex distribution was compared using the χ^2^ test. SD, standard deviation.

**Table 2:** Age distribution of the study population by disease severity.

Age Group (Years)	Moderate COVID-19, n	Severe COVID-19, n	Total, n (%)
18–39	3	7	10 (6.2%)
40–49	8	12	20 (12.1%)
50–59	20	25	45 (26.9%)
60–64	33	60	93 (54.8%)

Data are presented as number of patients and percentage of the total study population. Disease severity was classified according to the WHO Living Guidance for Clinical Management of COVID-19. Differences in age distribution between moderate and severe COVID-19 groups were assessed using the Chi-square test: χ^2^ = 1.41, df = 3, *p* = 0.704. χ^2^—chi-square statistic; df—degrees of freedom; *p*—*p*-value. A non-significant *p*-value indicates that the distribution of moderate and severe COVID-19 cases does not differ significantly across age groups.

**Table 3:** Descriptive characteristics of laboratory parameters in patients with moderate and severe COVID-19 on Day 1 (hospital admission) and Day 7 of hospitalization.

Variable	COVID-19	N	$\bar{x}$	Sx	$S\bar{x}$	Min	Max	*p*
Leukocytes Day 1	Moderate Severe All	64 104 168	8.36 8.43 8.40	2.29 2.27 2.27	0.29 0.22 0.18	4.59 4.59 4.59	15.60 17.60 17.60	0.838
Leukocytes Day 7	Moderate Severe All	64 104 168	7.34 7.41 7.38	1.65 1.62 1.63	0.21 0.16 0.13	4.37 4.22 4.22	10.51 10.94 10.94	0.795
Neutrophils (%) Day 1	Moderate Severe All	64 104 168	81.91 83.10 82.65	4.48 4.04 4.24	0.56 0.40 0.33	4.59 4.59 4.59	15.60 17.60 17.60	0.077
Neutrophils (%) Day 7	Moderate Severe All	64 104 168	79.83 80.35 80.15	4.80 4.47 4.59	0.60 0.44 0.35	70.10 70.10 70.10	88.50 93.80 93.80	0.479
Lymphocytes (%) Day 1	Moderate Severe All	64 104 168	10.34 10.00 10.13	3.07 2.85 2.93	0.38 0.28 0.23	3.80 1.90 1.90	16.10 16.80 16.80	0.473
Lymphocytes (%) Day 7	Moderate Severe All	64 104 168	12.20 11.89 12.01	2.72 2.77 2.75	0.34 0.27 0.21	5.60 3.60 3.60	17.90 17.50 17.90	0.484
NLR (%) Day 1	Moderate Severe All	64 104 168	10.34 10.00 10.13	3.07 2.85 2.93	0.38 0.28 0.23	3.80 1.90 1.90	16.10 16.80 16.80	0.469
NLR (%) Day 7	Moderate Severe All	64 104 168	7.05 7.26 7.18	2.35 2.42 2.39	0.29 0.24 0.18	4.04 4.07 4.04	15.57 16.05 16.05	0.578
Eosinophils (%) Day 1	Moderate Severe All	64 104 168	0.06 0.06 0.06	0.06 0.06 0.06	0.01 0.01 0.00	0.00 0.00 0.00	0.20 0.20 0.20	0.873
Eosinophils (%) Day 7	Moderate Severe All	64 104 168	0.13 0.12 0.12	0.04 0.05 0.05	0.01 0.01 0.00	0.10 0.00 0.00	0.20 0.20 0.20	0.620
Total protein (g/L) Day 1	Moderate Severe All	64 104 168	58.37 57.64 57.91	2.54 3.58 3.23	0.32 0.35 0.25	53.20 45.00 45.00	63.20 66.40 66.40	0.158
Total protein (g/L) Day 7	Moderate Severe All	64 104 168	58.85 58.18 58.44	2.74 3.71 3.38	0.34 0.36 0.26	53.60 45.00 45.00	66.30 67.40 67.40	0.216
Albumin (g/L) Day 1	Moderate Severe All	64 104 168	29.58 28.73 29.06	2.07 2.60 2.44	0.26 0.25 0.19	25.00 23.00 23.00	33.60 34.50 34.50	0.028
Albumin (g/L) Day 7	Moderate Severe All	64 104 168	29.20 28.32 28.65	2.33 3.05 2.83	0.29 0.30 0.22	24.00 23.00 23.00	34.80 34.60 34.80	0.049
ASAT (U/L) Day 1	Moderate Severe All	64 104 168	41.64 45.38 43.95	19.56 30.93 27.14	2.45 3.05 2.10	21.00 19.00 19.00	161.00 220.00 220.00	0.388
ASAT (U/L) Day 7	Moderate Severe All	64 104 168	26.33 33.09 30.50	12.63 38.55 31.38	1.58 3.80 2.43	11.00 8.00 8.00	102.00 322.00 322.00	0.503
ALAT (U/L) Day 1	Moderate Severe All	64 104 168	32.09 45.95 40.64	14.91 89.13 70.79	1.86 8.78 5.48	14.00 10.00 10.00	109.00 889.00 889.00	0.220
ALAT (U/L) Day 7	Moderate Severe All	64 104 168	26.32 33.09 30.50	12.63 38.55 31.38	1.58 3.80 2.43	11.00 8.00 8.00	102.00 322.00 322.00	0.177
GGT (U/L) Day 1	Moderate Severe All	64 104 168	39.58 43.59 42.05	26.85 37.91 34.06	3.36 3.74 2.64	14.00 16.00 14.00	192.00 334.00 334.00	0.461
GGT (U/L) Day 7	Moderate Severe All	64 104 168	38.17 39.26 38.84	48.29 42.94 44.93	6.04 4.23 3.48	13.00 15.00 13.00	383.00 392.00 392.00	0.879
Creatinine (µmol/L) Day 1	Moderate Severe All	64 104 168	94.30 120.30 110.34	15.57 115.08 91.60	1.95 11.34 7.09	63.00 63.00 63.00	134.00 853.00 853.00	0.074
Creatinine (µmol/L) Day 7	Moderate Severe All	64 104 168	86.14 97.09 92.89	13.44 56.48 45.36	1.68 5.57 3.51	62.00 60.00 60.00	121.00 459.00 459.00	0.130
CRP (mg/L) Day 1	Moderate Severe All	64 104 168	90.40 138.01 119.76	50.85 70.04 67.34	6.36 6.90 5.21	21.00 20.30 20.30	219.00 286.00 286.00	<0.0001
CRP (mg/L) Day 7	Moderate Severe All	64 104 168	57.81 100.20 84.05	35.46 68.00 61.26	4.43 6.67 4.73	10.10 12.60 10.10	142.60 353.00 353.00	<0.0001
LDH (U/L) Day 1	Moderate Severe All	64 104 168	864.84 1263.36 1111.54	219.55 763.42 644.45	27.44 74.86 49.72	578.00 54.00 54.00	1427.00 6294.00 6294.00	<0.0001
LDH (U/L) Day 7	Moderate Severe All	64 104 168	689.70 1026.99 898.50	194.78 528.80 462.35	24.35 51.85 35.67	405.00 363.00 363.00	1238.00 3507.00 3507.00	<0.0001
Ferritin (ng/mL) Day 1	Moderate Severe All	64 104 168	824.42 997.03 931.27	326.30 403.92 384.53	40.79 39.61 29.67	290.60 237.50 237.50	1534.00 2102.00 2102.00	0.004
Ferritin (ng/mL) Day 7	Moderate Severe All	64 104 168	935.75 1160.79 1075.06	314.11 470.69 431.14	39.26 46.16 33.26	445.20 178.30 178.30	1723.00 2378.10 2378.10	<0.001
IL-6 (pg/mL) Day 1	Moderate Severe All	64 104 168	31.90 51.72 44.17	22.50 32.76 30.76	2.81 3.21 2.37	10.00 13.93 10.00	102.00 132.22 132.22	<0.0001
IL-6 (pg/mL) Day 7	Moderate Severe All	64 104 168	27.33 53.88 43.76	16.84 38.25 34.30	2.10 3.75 2.65	10.61 11.15 10.61	80.60 164.53 164.53	<0.0001
PCT (ng/mL) Day 1	Moderate Severe All	64 104 168	0.62 0.89 0.79	0.18 0.42 0.37	0.02 0.04 0.03	0.33 0.36 0.33	1.15 1.94 1.94	<0.0001
PCT (ng/mL) Day 7	Moderate Severe All	64 104 168	0.62 0.88 0.78	0.56 0.53 0.56	0.07 0.05 0.04	0.32 0.31 0.31	4.77 1.99 4.77	0.003
D-Dimer (µg/mL) Day 1	Moderate Severe All	64 104 168	2.25 4.49 3.64	2.72 6.28 5.32	0.34 0.62 0.41	0.25 0.57 0.25	21.02 35.20 35.20	0.008
D-Dimer (µg/mL) Day 7	Moderate Severe All	64 104 168	3.29 6.27 5.13	6.17 8.38 7.73	0.77 0.82 0.60	0.49 0.22 0.22	35.20 35.20 35.20	0.015
Vitamin D (ng/mL) Day 1	Moderate Severe All	64 104 168	17.40 15.65 16.31	4.37 4.11 4.28	0.55 0.40 0.33	8.38 5.45 5.45	29.68 26.18 29.68	0.010
Vitamin D (ng/mL) Day 7	Moderate Severe All	64 104 168	20.83 19.03 19.71	3.81 3.32 3.61	0.48 0.33 0.28	13.24 12.25 12.25	32.02 28.74 32.02	<0.001
sO_2_ (%) Day 1	Moderate Severe All	64 104 168	89.16 85.12 87.93	8.44 8.63 8.21	0.41 0.44 0.33	76.00 62.00 62.00	91.00 88.00 91.00	<0.001
sO_2_ (%) Day 7	Moderate Severe All	64 104 168	95.78 90.15 95.36	3.42 3.54 3.16	0.39 0.36 0.24	95.00 65.00 65.00	98.00 95.00 95.00	<0.001

Data are presented as mean ($\bar{x}$), standard deviation (Sx), standard error of the mean (S$\bar{x}$), and range (minimum (Min)—maximum (Max)). N indicates the number of patients with available measurements for each parameter. Comparisons between groups were performed using Student’s *t*-test or the Mann–Whitney U test, as appropriate, based on data distribution. A two-sided *p* value < 0.05 was considered statistically significant. CRP, C-reactive protein; LDH, Lactate dehydrogenase; IL-6, interleukin-6; PCT, procalcitonin.

**Table 4:** Pairwise Pearson correlation and Variance Inflation Factor (VIF) values for inflammatory biomarkers at hospital admission (Day 1).

Biomarker	CRP	IL-6	Ferritin	PCT	VIF
CRP	1.00	0.62	0.55	0.48	2.1
IL-6	0.62	1.00	0.51	0.44	2.4
Ferritin	0.55	0.51	1.00	0.39	1.9
PCT	0.48	0.44	0.39	1.00	2.2

Pearson correlation coefficients (r) were calculated for laboratory values obtained at hospital admission (Day 1) (*n* = 168). All correlation coefficients were below the threshold indicating high collinearity (r > 0.80). Variance inflation factor (VIF) values < 3 indicate the absence of problematic multicollinearity. CRP, C-reactive protein; IL-6, interleukin-6; PCT, procalcitonin.

**Table 5:** Independent predictors of in-hospital mortality in patients with severe COVID-19 at hospital admission (Day 1): multivariable Cox regression analysis.

Predictor	Coefficient (B)	SE	*p*-Value	HR	95% CI for HR
Age (years)	0.091	0.030	0.003	1.10	1.03–1.16
Sex (male vs. female)	−1.126	0.451	0.013	0.32	0.13–0.79
Lymphocytes (%)	−0.140	0.068	0.041	0.87	0.76–0.99
PCT (ng/mL)	2.395	0.530	<0.001	10.97	3.87–30.98

Values represent results from a multivariable Cox proportional hazards model including patients with severe COVID-19 (*n* = 104). B, regression coefficient; SE, standard error; HR, hazard ratio; CI, the 95% confidence interval. HR > 1 indicates increased hazard of in-hospital death, whereas HR < 1 indicates decreased hazard.

**Table 6:** Independent predictors of in-hospital mortality in all patients with COVID-19 at hospital admission (Day 1): multivariable Cox regression analysis.

Predictor	Coefficient (B)	SE	*p*-Value	HR	95% CI for HR
Age (years)	0.095	0.031	0.002	1.10	1.04–1.17
Sex (male vs. female)	−1.197	0.450	0.008	0.30	0.13–0.73
Lymphocytes (%)	−0.148	0.069	0.030	0.86	0.75–0.99
PCT (ng/mL)	2.705	0.511	<0.001	14.95	5.49–40.74

Values represent results from a multivariable Cox proportional hazards model including all COVID-19 patients (*n* = 168). B, regression coefficient; SE, standard error; HR, hazard ratio; CI, the 95% confidence interval.

**Table 7:** Independent predictors of in-hospital mortality in patients with severe COVID-19 on Day 7 of hospitalization: multivariable Cox regression analysis.

Predictor	Coefficient (B)	SE	*p*-Value	HR	95% CI for HR
Sex (male vs. female)	−1.145	0.547	0.036	0.32	0.11–0.93
PCT (ng/mL)	4.308	0.914	<0.001	74.27	12.38–445.70
Eosinophils (%)	−13.481	6.269	0.032	0.001	0.001–0.303
Ferritin (ng/mL)	0.002	0.001	0.025	1.002	1.000–1.003
Vitamin D (ng/mL)	−0.153	0.085	0.071	0.86	0.73–1.01
GGT (U/L)	0.017	0.005	0.001	1.02	1.01–1.03

Values represent results from a multivariable Cox proportional hazards model including patients with severe COVID-19 (*n* = 104). B, regression coefficient; SE, standard error; HR, hazard ratio; CI, the 95% confidence interval. HR > 1 indicates increased hazard of in-hospital death, whereas HR < 1 indicates decreased hazard.

**Table 8:** Independent predictors of in-hospital mortality in all patients with COVID-19 on Day 7 of hospitalization: multivariable Cox regression analysis.

Predictor	Coefficient (B)	SE	*p*-Value	HR	95% CI for HR
Age (years)	0.070	0.023	0.002	1.07	1.03–1.12
PCT (ng/mL)	1.490	0.332	<0.001	4.44	2.31–8.51
IL-6 (pg/mL)	0.026	0.007	<0.001	1.03	1.01–1.04
GGT (U/L)	0.012	0.004	0.001	1.01	1.01–1.02

Values represent results from a multivariable Cox proportional hazards model including all COVID-19 patients (*n* = 168). B, regression coefficient; SE, standard error; HR, hazard ratio; CI, the 95% confidence interval.

**Table 9:** Predictors associated with in-hospital mortality across four multivariable Cox regression models.

Predictor	Severe COVID-19 (Day 1)	All COVID-19 (Day 1)	Severe COVID-19 (Day 7)	All COVID-19 (Day 7)
Age (years)	Significant	Significant	Not significant	Significant
Sex (male vs. female)	Significant	Significant	Significant	Not significant
Lymphocytes (%)	Significant	Significant	Not significant	Not significant
Eosinophils (%)	Not significant	Not significant	Significant	Not significant
PCT (ng/mL)	Significant	Significant	Significant	Significant
Ferritin (ng/mL)	Not significant	Not significant	Significant	Not significant
IL-6 (pg/mL)	Not significant	Not significant	Not significant	Significant
Vitamin D (ng/mL)	Not significant	Not significant	Significant	Not significant
GGT (U/L)	Not significant	Not significant	Significant	Significant

“Significant” indicates *p* < 0.05 in the corresponding multivariable Cox proportional hazards regression model. All models were adjusted for covariates retained through stepwise selection.

**Table 10:** Assessment of the proportional hazards assumption for the Cox regression models.

Model	Global χ^2^	Global *p*-Value	Interpretation
Severe COVID-19—Day 1	3.42	0.49	No PH violation
Severe COVID-19—Day 7	5.11	0.28	No PH violation
All patients—Day 1	2.87	0.58	No PH violation
All patients—Day 7	4.02	0.40	No PH violation

Global chi-square (χ^2^) statistics and corresponding *p*-values were obtained from tests based on Schoenfeld residuals. No covariate-specific test showed significant deviation from the proportional hazards assumption (all *p* > 0.05).

**Table 11:** Receiver operating characteristic (ROC) performance of prognostic Cox regression models for in-hospital mortality.

Patient Group	AUC	SE	*p*
Severe COVID-19 Day 1	0.895	0.036	<0.001
Severe COVID-19 Day 7	0.957	0.022	<0.001
All COVID-19 Day 1	0.901	0.035	<0.001
All COVID-19 Day 7	0.952	0.021	<0.001

AUC, area under the receiver operating characteristic curve; SE, standard error. *p*-values indicate comparisons of AUC values against the null hypothesis of no discriminative ability (AUC = 0.5).

**Table 12:** Optimal cut-off values, sensitivity, and specificity derived using the Youden index for prognostic models predicting in-hospital mortality.

Patient Group	AUC	Optimal Cut-Off (Youden)	Sensitivity	Specificity
Severe COVID-19 Day 1	0.895	0.38	0.84	0.81
Severe COVID-19 Day 7	0.957	0.41	0.92	0.90
All COVID-19 Day 1	0.901	0.36	0.86	0.80
All COVID-19 Day 7	0.952	0.40	0.91	0.89

Optimal cut-off values were determined using the Youden index (J = sensitivity + specificity − 1). Sensitivity and specificity were calculated at the threshold corresponding to the maximum Youden index for each model. AUC, area under the receiver operating characteristic curve.

## Data Availability

All data generated or analyzed during this study are included in this published article.
